# The 3D skull 0–4 years: A validated, generative, statistical shape model

**DOI:** 10.1016/j.bonr.2021.101154

**Published:** 2021-11-29

**Authors:** Eimear O' Sullivan, Lara S. van de Lande, Anne-Jet C. Oosting, Athanasios Papaioannou, N. Owase Jeelani, Maarten J. Koudstaal, Roman H. Khonsari, David J. Dunaway, Stefanos Zafeiriou, Silvia Schievano

**Affiliations:** a Great Ormond Street Institute of Child Health, University College London & Craniofacial Unit, Great Ormond Street Hospital for Children, London, UK; b Department of Computing, Imperial College London, London, UK; c Department of Oral and Maxillofacial Surgery, Erasmus Medical Centre, Rotterdam, the Netherlands; d Oral and Maxillofacial Surgery Department, Hospital Necker, Enfants Malades, Paris, France

**Keywords:** Paediatric skull, Morphometrics, Statistical shape model, 3D morphable model, Synthetic shapes

## Abstract

**Background:**

This study aims to capture the 3D shape of the human skull in a healthy paediatric population (0–4 years old) and construct a generative statistical shape model.

**Methods:**

The skull bones of 178 healthy children (55% male, 20.8 ± 12.9 months) were reconstructed from computed tomography (CT) images. 29 anatomical landmarks were placed on the 3D skull reconstructions. Rotation, translation and size were removed, and all skull meshes were placed in dense correspondence using a dimensionless skull mesh template and a non-rigid iterative closest point algorithm. A 3D morphable model (3DMM) was created using principal component analysis, and intrinsically and geometrically validated with anthropometric measurements. Synthetic skull instances were generated exploiting the 3DMM and validated by comparison of the anthropometric measurements with the selected input population.

**Results:**

The 3DMM of the paediatric skull 0–4 years was successfully constructed. The model was reasonably compact - 90% of the model shape variance was captured within the first 10 principal components. The generalisation error, quantifying the ability of the 3DMM to represent shape instances not encountered during training, was 0.47 mm when all model components were used. The specificity value was <0.7 mm demonstrating that novel skull instances generated by the model are realistic. The 3DMM mean shape was representative of the selected population (differences <2%). Overall, good agreement was observed in the anthropometric measures extracted from the selected population, and compared to normative literature data (max difference in the intertemporal distance) and to the synthetic generated cases.

**Conclusion:**

This study presents a reliable statistical shape model of the paediatric skull 0–4 years that adheres to known skull morphometric measures, can accurately represent unseen skull samples not used during model construction and can generate novel realistic skull instances, thus presenting a solution to limited availability of normative data in this field.

## Highlights

• 3D morphable model of the paediatric skull (0–4 years) was created from 178 CT scans.
• The model was validated using compactness, generalisation and specificity.
• Validation experiments demonstrated that the model can produce realistic novel skull samples.
• Skull shape was shown to be a good indicator of age for the study population.
• Gender was not shown to play a role in skull shape for the study population.

## 1 Introduction

In silico medicine refers to the development and use of computational models that realistically mimic and simulate patients' biology and medical interventions in a virtual environment (Pappalardo et al., 2019). These models aim at explaining diseases, supporting diagnosis and prevention, and predicting treatment outcomes, with the advantage that several therapy options and approaches can be simulated in the same virtual patient to personalise and optimise results. In silico medical modelling can complement medical device/drug development by reducing, refining or partially replacing the three traditional sources of evidence - bench testing, animal testing and human clinical trials - to establish device/drug safety and effectiveness, at significantly lower costs (Viceconti et al., 2021). This can be of particular benefit for those medical fields that focus on rare diseases, such as craniofacial syndromes, where availability of data is limited, relevant animal models are lacking, enrolling large cohorts of real patients is unfeasible and the market is overall too small for the medical device/pharma industry to heavily invest in R&D.

To be valid and effective, in silico clinical trials still relay on cohorts of real, clinical data (Badano, 2021). However, collecting and annotating large quantities of data to train and test the computational models is expensive and time-consuming, thus restricting the choice of evaluation and modelling algorithms that can be applied. Ethical considerations further limit the purposes for which patient datasets can be used. Conversely, generated synthetic data are easy to manipulate, fully user controlled and carry limited ethical concerns. The ability to generate new samples means that cohort size is not a limiting factor, overcoming statistical power issues and the burden of enrolling large cohorts of real patients (Faris and Shuren, 2017).

3D morphable models (3DMMs) use advanced statistical methods applicable to a population of computational shapes to study and describe the population's complex 3D shape features. First proposed by Blanz and Vetter (Blanz and Vetter, 1999; Booth et al., 2016) in 1999, 3DMMs have proven adept at modelling complex shape structures such as the human face, head, hand and ear (Blanz and Vetter, 1999; Booth et al., 2016; Dai et al., 2018; Ploumpis et al., 2019; Dai et al., 2017; Zolfaghari et al., 2016; Khamis et al., 2015) despite limited input data. Paediatric skull models have also been constructed using 3DMMs, although the ability of these models to generate synthetic data has not yet been tested (Li et al., 2015; Kuwahara et al., 2020; Libby et al., 2017).

In this study, we present a scale-normalised 3DMM of the normal paediatric skull, 0–4 years of age, that can be used to generate novel synthetic skull shapes. The 3DMM is validated using both intrinsic 3DMM characteristics and extrinsic anthropometric measures.

## 2 Materials and methods

### 2.1 Data and image processing

The study cohort included 178 children (55% male) aged between 0 and 48 months at time of computerised tomography (CT) scanning (20.3 ± 12.9 months). All CT scans were acquired at Necker Enfants Malades Hospital, Assistance Publique – Hôpitaux de Paris, Paris, France between 2011 and 2018, with indications for headache, epilepsy, or trauma assessment. Scans were considered eligible with DICOM-slices of ≤1 mm with a minimum 150 slices. Two independent reviewers retrospectively checked the scans to include only studies which presented no structural abnormalities – no brain and bone tumours, skull fractures, or craniofacial anomalies – and with sufficient quality – no movement artefacts or low number of slices.

**Fig. 1:**
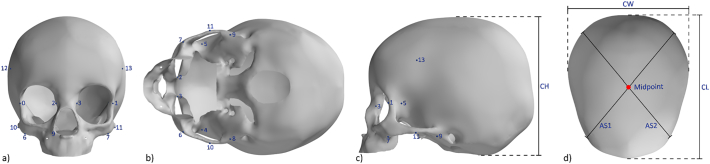
Template skull mesh. Landmarks and distance definitions for the calculation of anthropometric skull measurements (Waitzman et al., 1992) from the frontal, inferior, sagittal and top view. CH = cranial height; CW = cranial width; CL = cranial length; AS = transcranial length.

The skull anatomy was reconstructed from the CT scans using thresholding in Mimics InPrint 3.0 (Materialise NV, Leuven, Belgium) and the generated mesh exported for further processing. A dimensionless mesh template of fixed topology was used to place all skulls in dense correspondence. The skull meshes were first rigidly aligned with the mesh template using Procrustes registration before dense correspondence was achieved using a non-rigid iterative closest point algorithm (Amberg et al., 2007). A set of 29 anatomical landmarks was used for the rigid alignment and to guide the non-rigid iterative closest point process (Supplementary Material Table 1 and Fig. 1). The dataset included many samples with an open anterior fontanelle and varying levels of suture closure, as expected at this age. Thus, a “stiffness” factor was added to the template mesh in the region of the opening to facilitate the registration process and prevent inwards collapse of the template mesh in this region (Supplementary Material Fig. 2).

The anthropometric linear measures by Waitzman et al. (1992) listed in Table 1 were automatically extracted for all reconstructed skull geometries using a new set of landmarks on the 3D template, as shown in Fig. 1. The Euclidean distance between given landmarks were computed, the mean and standard deviation for the population were calculated and the population mean size was added back to the measures for comparison with Waitzman et al. Supplementary Material provides information on how the mean and standard deviation were collated from the work of Waitzman et al.

**Table 1:** Anthropometric measures and definitions (Waitzman et al., 1992), with numbers referring to Fig. 1. R = right side; L = left side.

Landmark	Anthropometric measure	Description
0–1	Lateral orbital distance	Distance between the mid-lateral points of the lateral orbital walls.
2–3	Anterior inter-orbital distance	Distance between the anterior points of the medial orbital walls, measured from 2 points that are on the same axial level as points 0–1.
4–5	Inter-temporal distance	Distance between the most concave point on each temporalis groove. The points are on the same axial level as points 0–3.
6–7	Inter-zygomatic buttress distance	Distance between the most anterolateral corners of each zygomatic buttress.
6–8 (R) 7–9 (L)	Zygomatic arch length R/L	Distance between the most anterolateral corner of the zygomatic buttress and the insertion of the zygomatic arch into the squamous part of the temporal bone of the skull, placed on the most inferior point.
10–11	Inter-zygomatic arch distance	Distance between the most convex points on each zygomatic arch.
12–13	Inter-coronal distance	Distance between the 2 most lateral points on the coronal suture.

The skull meshes, aligned with the template, were placed in a straight, forward-facing position, to extract automatically the following traditional 2D morphometric skull distances: cephalic length (CL), width (CW), height (CH) and asymmetric transcranial lengths (AS1 and AS2). These measures were used to calculate the skull cephalic index (CI) as 100 ∗ (CW/CL), height index (HI) as 100 ∗ (CH/CL) and oblique cranial length ratio (OCLR) as the ratio of the longer to the shorter transcranial length. The mean and standard deviations of the defined lengths and ratios were computed for the population and compared with those reported by Waitzman et al.

### 2.2 3D morphable model

The 3D mean anatomical skull shape for the population was computed based on all the densely registered meshes. PCA was applied to build the 3DMM and detect key contributors to 3D shape variability in the population. The first 5 principal components, representing the key 3D shape features of the input population, were visualised as −3SD to +3SD deformations from the mean shape.

The 3DMM mean shape was validated via a 10-fold cross validation approach – to assure that the final mean shape was not overly influenced by adding or leaving out a specific subject – and using a geometric approach – to demonstrate that the 3D mean shape was representative of the study cohort (Bruse et al., 2017). For the 10-fold cross validation, the study population was divided randomly into 10 subsets or folds. The mean shape was then computed using 9 of the folds, until each fold had been omitted once. The mean and standard deviation vertex difference between the original 3D mean shape and cross validated shapes was then calculated. For the geometrical approach, the distances from Waitzman et al. (Table 1) and the additional 2D morphometric lengths and ratios measured from the 3DMM mean shape were compared to the mean values calculated from the input population; deviations <5% were considered acceptable for the mean shape to represent the population with a good approximation.

The intrinsic characteristics of 3DMMs were evaluated with compactness, generalisation and specificity, as done in the literature (Blanz and Vetter, 1999; Booth et al., 2016). For compactness, the percentage of shape variation explained by the model was plotted versus the respective number of retained principal components. For generalisation – a measure of the model ability to represent novel shape instances not encountered during training – given the relatively small sample size, a leave-one-out strategy was adopted. To calculate the generalisation error for a sample in the test set at a given number of model principal components, the average Euclidean distance (AED) between the sample and its corresponding model projection was computed on a per-vertex basis:

$$\mathrm{AED}=\frac{\sum_{i=1}^{n}\sqrt{{\left(x_{i,A}-x_{i,B}\right)}^{2}+{\left(y_{i,A}-y_{i,B}\right)}^{2}+{\left(z_{i,A}-z_{i,B}\right)}^{2}\;}}{n}$$

with *n* = number of mesh vertices, and *x*, *y*, and *z* = Cartesian coordinates of meshes *A* and *B*. The overall generalisation error at each principal component was then calculated as the mean per-vertex error over all meshes. For model specificity, which evaluates the validity of novel instances generated by the 3DMM, a leave-one-out system was also adopted. To measure the model specificity, 1000 synthetic skulls were randomly generated for each of the model principal components. The distance between each synthetic skull and its nearest neighbour in the test set was then calculated as the average Euclidean distance over all mesh vertices.

Using the 3DMM, additional 1000 skull samples were randomly generated using all principal components. Meshes were synthesised by drawing shape vectors at random from a multivariate Gaussian distribution about the model principal components. Traditional anthropometric linear measures (Table 1 and additional lengths and rations) were extracted automatically as explained before for each of the synthesised skull samples, and mean and standard deviation of each of this metrics from the synthetic population were compared to those from the input population for validation purposes, using a two-tailed *t*-test. A value of 0.05 was considered for statistical significance.

### 2.3 Age and gender based shape changes

For each of the registered skull meshes, the corresponding shape parameters were determined by projecting the skull instance into the latent shape space of the PCA model. This yielded a set of shape vectors, α = [*α*_1_, *α*_2_, …, *α*_*n*_], where *n* is the number of meshes and each *α*_*i*_ is a representation of the corresponding mesh instance in the high-dimensional latent space of the PCA model. These shape vectors were then used to assess how age and gender influence the skull shape in the study cohort. A ten-fold cross validation schema was used in both cases.

**Fig. 2:**
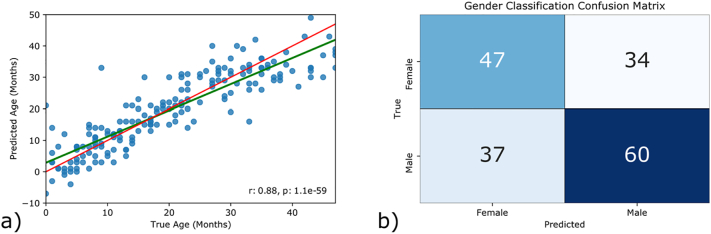
a) Correlation between predicted age in months and age at time of scan when all 10 PLS modes were used for regression during a 10-fold cross-validation. The green line indicates the line of best fit between the ground truth data and the prediction. The red line is the line of equality. b) Confusion matrix for gender prediction during a 10-fold cross-validation.

When the shape vectors were used for linear age regression, a root mean square error (RMSE) of 6.1 months and an R2 score of 0.77 were observed (Fig. 2). These results indicate that age can be inferred from skull shape with a reasonable degree of accuracy. Shape proved to be a poor predictor of gender, however, and a support vector machine trained to classify instances as either male of female achieved an accuracy of 60.1% (Fig. 2). Due to the uneven gender distribution, this is only slightly better than the maximum chance accuracy of 54.5%, and it is unlikely that this can be attributed to differences in skull shape.

## 3 Results

The mean and standard deviation of the selected population for the extracted anthropometric measures are shown in Table 2 where they are compared with the data from Waitzman et al. (1992), showing similar values and thus validating the selected cohort as representative of the 0–4-year normative population. The greatest difference between the two datasets was observed for the intertemporal distance: as growth in the skull region is rapid for children in the assessed age range, some of the measurement differences may be attributed to the different age distributions of the two study populations (Supplementary Materials).

**Table 2:** Skull anthropometric measures reported by Waitzman et al. (1992), and extracted from the selected population, from the 3DMM mean shape and from the generated synthetic cases.

Anthropometric measure	Waitzman et al. [*n* = 166]	Population [*n* = 178]	Mean shape (% deviation from population mean)	Synthetic samples (population vs. synthetic *p* values) [*n* = 1000]
Lateral orbital distance	74.18 ± 5.71	78.12 ± 5.33	78.77 (−0.83)	78.96 ± 3.54 (0.044)
Anterior inter orbital distance	18.30 ± 1.93	15.91 ± 1.52	16.06 (−0.92)	16.16 ± 1.52 (0.046)
Intertemporal distance	64.80 ± 5.21	75.97 ± 5.24	76.61 (−0.84)	76.77 ± 3.80 (0.054)
Inter-zygomatic buttress distance	70.20 ± 6.35	71.73 ± 6.58	72.14 (−0.57)	72.25 ± 2.25 (0.296)
Zygomatic arch length	42.64 ± 4.78	38.04 ± 4.76	38.05 (−0.03)	38.05 ± 1.88 (0.982)
Inter-zygomatic arch distance	86.86 ± 8.07	92.75 ± 8.82	93.24 (−0.53)	93.34 ± 2.64 (0.379)
Inter-coronal distance	101.11 ± 8.87	104.53 ± 8.42	105.31 (−0.74)	105.49 ± 5.09 (0.143)
CL	157.79 ± 14.82	151.77 ± 15.73	152.01 (−0.15)	152.35 ± 6.51 (0.631)
CW	121.93 ± 10.09	128.43 ± 10.60	128.38 (0.04)	129.79 ± 6.71 (0.102)
CH	–	131.05 ± 11.24	130.56 (0.37)	132.18 ± 5.03 (0.187)
AS1	–	143.24 + − 12.41	143.889 (−0.453)	144.36 ± 5.60 (0.239)
AS2	–	141.85 + − 12.53	142.232 (−1.437)	142.75 ± 5.60 (0.346)
CI	–	85.04 ± 6.34	84.46 (0.68)	85.38 ± 6.25 (0.500)
HI	–	86.60 ± 4.25	85.89 (0.82)	86.88 ± 4.16 (0.422)
OCLR	–	1.03 ± 0.03	1.01 (2.00)	1.03 ± 0.03 (0.503)

**Fig. 3:**
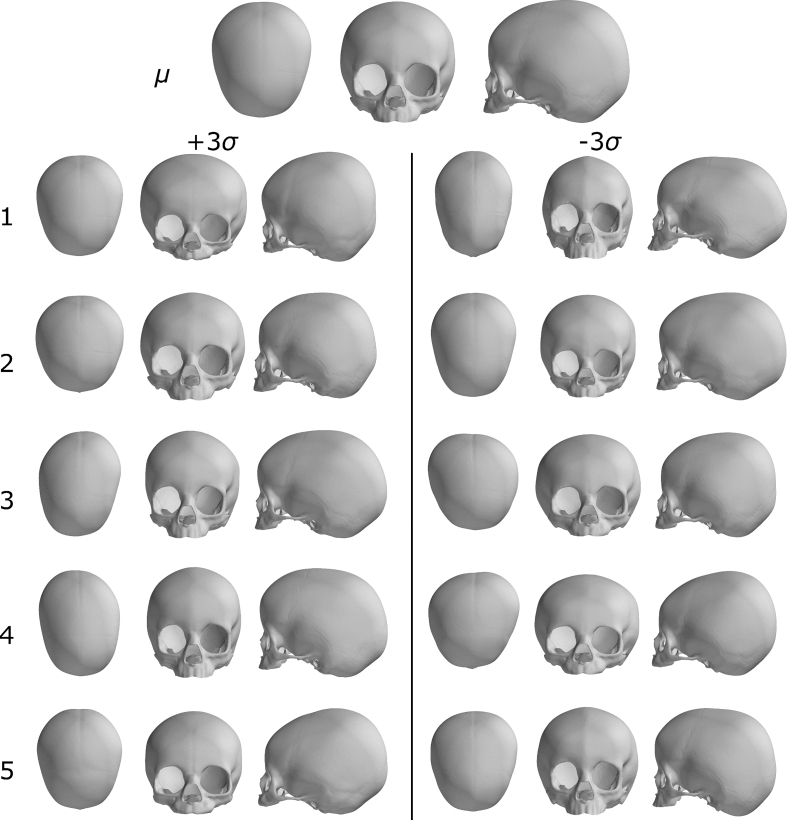
Visualisation of the paediatric skull model: mean shape, μ, and first five principal components, visualised as either an addition or a subtraction from the mean shape with a weight of ±3σ, where σ_i_ is the standard deviation of the i-th principal component. Each model instance is shown from the top, frontal and sagittal view.

**Fig. 4:**
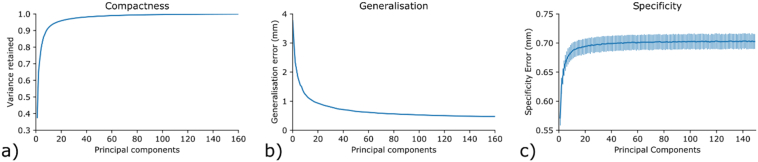
a) Compactness; b) generalisation; and c) specificity of the paediatric skull model.

The mean shape and the first five principal modes of shape variation are shown in Fig. 3 from three different viewpoints: aerial, front, and side. The first principal component, accounting for 37.58% of the shape variation in the population (Fig. 4a), captures clear differences in overall skull length, width and height, from a globally rounded shape to a more elongated in the antero-posterior direction, narrower and less tall in the posterior portion skull. The second component highlights shape variations localised mainly in the frontal portion of the skull, with differences in midfacial width compared to forehead height proportion, and orbital size, albeit changes in skull length are also still visible. The third and fourth components show asymmetrical shape differences in the posterior portion of the skull. The fifth component highlights more subtle differences in the skull height anterior/posterior proportions, orbital size and shape, and protrusion of the mid-face.

The 10-fold cross-validation confirmed that removing arbitrary subjects from the study population did not significantly impact the computed mean shape: the mean vertex distance between original mean shape and cross validated shapes was 0.13 ± 0.10 mm. The computed 3DMM mean shape showed good agreement with the mean of the study population across all parameters (Table 2). The only percentage difference between the cohort mean and the computed mean shape >1% was observed for OCLR (2.0%), indicating that the computed mean shape is slightly more symmetric in shape that the population average. Thus, the 3DMM mean shape was considered validated and representative for the study cohort.

Intrinsic model characteristics are shown in Fig. 4. With 90% of the model shape variance captured within the first 10 principal components, and 95% of the variance captured within the first 20 principal components (Fig. 4a), the model was considered reasonably compact. The generalisation error was 1 mm when 15 principal components were included, and 0.47 mm when all model components were utilised (Fig. 4b), indicating that the model generalised well to unseen skull instances. Specificity values of less than 0.7 mm (Fig. 4c) demonstrated that novel skull instances generated by the model are realistic.

The measures from the synthetic cohort (*n* = 1000) generated using all 3DMM components show good agreement with the corresponding values for the study cohort. The two-tailed *t*-test indicates that the null hypothesis of equal means can be accepted for all parameters, apart from the lateral orbital distance and the anterior inter orbital distance (Table 2). The mean values for both real and synthetic data were very close, however, and differences between the real and synthetic populations can likely be attributed to the difficulty of accurately and consistently localising these landmarks. Smaller standard deviations were observed for the generated samples; this can likely be attributed to the omission of size effects from the model.

## 4 Discussion

This study presents a 3DMM of the paediatric skull (0–4 years), constructed from 178 normal CT scans. The model is a 3D reference of the normative skull shape for this age population and can be used to generate valid paediatric skull instances, as an alternative to the limited availability of CT images from healthy children, thus opening doors for many applications where access to data is currently a hurdle.

Human head size and shape are constantly developing throughout lifetime due to numerous anatomical and functional factors including skeletal growth, brain and sinus development, and airway volume increase, particularly pronounced during early childhood. The pace of these skeletal changes, coupled with the difficulties of CT scanning healthy children due to the risks associated to ionizing radiations, makes collection of normative 3D skull data challenging for this group of young age individuals. Current craniomaxillofacial clinical practice leverages normative 2D facial anthropometric and cephalometric measurements and growth curves from literature, such as those presented in the seminal work by Waitzman et al. - the most comprehensive literature available on craniofacial skeletal measures in a paediatric healthy population - to assess deviation from normal, diagnose craniofacial diseases, and plan and evaluate treatment outcomes. However, these approaches remain limited as based on 2D and linear measurements between spare anatomical landmarks that cannot capture the full complexity of the head shape in 3D. Conversely, 3DMMs such as that presented in this study, can automatically transfer hundreds of corresponding landmarks from a generic template onto each 3D subject-specific skull reconstruction, thus rapidly extracting full 3D information on the individual patient skull shape, and fostering advanced 3D analysis and in silico medicine.

To validate the proposed 3DDM, the healthy cohort selected was compared to the normative population presented by Waitzman et al. (1992). Traditional anthropometric linear measures extracted from the selected population compared well with those published by Waitzman et al., showing that the selected group was a good representation of the 1–4-year-old healthy population. The 3DMM principal components, independent of the overall head growth, described the main shape variations encountered in the population, capturing overall vault shape changes, but also more subtle differences in the skeletal structure of the face. The 3DMM and the ability to generate synthetic skull instances were validated both methodologically (compactness, generalisation, and specificity) and by comparison of the results (3DMM mean shape and synthetised shapes) with the anthropometric measures of the input population. A strong agreement on all assessed skull measures was observed in both cases, thus confirming that the constructed 3DMM is robust, captures average ‘expected’ skull shapes and statistical variations in the given population, and can be useful to generate valid synthetic cases.

Using this normative 3D model, the skull CT data from new patients could be automatically compared to evaluate normal and abnormal skull shape features, for diagnostic purposes and to potentially plan personalised surgical treatments of craniomaxillofacial syndromes when needed (Farkas et al., 1992; Mauler et al., 2017; Knoops et al., 2019; Staal et al., 2015; Fuessinger et al., 2018). Craniofacial injuries due to falls, common among young children, and various craniofacial defects could be treated using automatically designed implants, by leveraging synthetic skull data to train the design algorithm, thus making the process less operator dependent and time consuming (Li et al., 2021). To facilitate the development of new model analysis techniques and clinical treatments by research groups without direct access to medical data, the normative 3DMM and synthetically generated new instances could support in silico medicine and clinical trials for the design of surgical devices and implants. Non-medical industries could also benefit from the normative paediatric skull 3DMM, for example for smart development of more protective safety helmets and for building more relevant dummies to facilitate accurate simulation and testing of car accidents.

With an eye on future use and model improvement, the authors initiated the construction of a mandible model and aim to combine the proposed skull model with soft tissue 3DMMs of the face. This may contribute to refining surgical planning tools, more powerful craniofacial diagnosis algorithms and postoperative outcome assessment.

## 5 Conclusions

A paediatric 3D skull model based on normative data was presented and holds future applications for the synthesis of novel skull instances which may have value for in silico medical applications. In medical studies where limited patient data is available, this model has the potential to help achieve statistical power by providing an unlimited data source that is free of ethical requirements, flexible, and easy to manipulate. It can assist in the development and support the design of surgical instruments and medical devices, such as helmet shapes for those with a distorted head shape.

## Credit authorship contribution statement

Ms. Eimear O' Sullivan contributed to the conception, design, analysis, interpretation of the data, writing and revision of the manuscript. Dr. Lara S. van de Lande contributed to the design, acquisition and analysis of the data, interpretation of the data, writing and revision of the manuscript. Ms. Anne-Jet C. Oosting contributed to the analysis of the data and critical analysis of the manuscript. Dr. Athanasios Papaioannou contributed to the design, data analysis and critical analysis of the manuscript. Mr. N. Owase Jeelani and Dr. Maarten J. Koudstaal, contributed to the critical analysis of the manuscript. Prof. Roman H. Khonsari contributed to the data acquisition and critical analysis of the manuscript. Prof. Silvia Schievano, Prof. David J. Dunaway, and Prof. Stefanos Zafeiriou contributed to the conception, design, interpretation of the data, and critical analysis of the manuscript.

## Declaration of competing interest

A.P. and S.Z. currently work with Huawei Technologies Co., Ltd. They were with University College London and Imperial College London during the experiments, respectively. The other authors declare no competing interests.

## Appendix A Supplementary data

Model construction and analysis code and 3D assets. Image 1

upplementary figures, tables, and algorithms. Image 2
