# Supplementary material for: The 3D skull 0–4 years: A validated, generative, statistical shape model
Source: Bone Rep. 2021 Nov 29;15:101154. doi: 10.1016/j.bonr.2021.101154 (PMC8645852; doi:10.1016/j.bonr.2021.101154)
Supplement: Supplementary file 2 — upplementary figures, tables, and algorithms. [file mmc2.docx]

**The 3D skull 0-4 years: a validated, generative, statistical shape model**

Eimear O’ Sullivan^†^ (1,2), Lara S. van de Lande^†^ (1), Anne-Jet C. Oosting (1,3), Athanasios Papaioannou (1,2), N. Owase Jeelani (1), Maarten J. Koudstaal (3), Roman H. Khonsari (4), David J. Dunaway (1), Stefanos Zafeiriou (2), Silvia Schievano (1)

† These authors contributed equally to this work.

1. Great Ormond Street Institute of Child Health, University College London & Craniofacial Unit, Great Ormond Street Hospital for Children, London, UK

2. Department of Computing, Imperial College London, London, UK

3. Department of Oral and Maxillofacial Surgery, Erasmus Medical Centre, Rotterdam, the Netherlands

4. Oral and Maxillofacial Surgery Department, Hospital Necker, Enfants Malades, Paris, France

**Corresponding author:**

Silvia Schievano

The Zayad Centre for Research

20 Guilford St

London, WC1N 1DZ

s.schievano@ucl.ac.uk

# **Supplementary Material**

## **Landmark Definitions**

| Landmark number | Landmark name | Definition |
| --- | --- | --- |
| 0 | Nasion | Midpoint of the nasalfrontal suture |
| 1 | Anterior nasal spine (ANS) | Most anterior mid-point of the pointed projection formed by the protrusion of the maxilla at the nose base |
| 2 | A point | Point of the maximum concavity anteriorly of the maxillary alveolar process in the midline |
| 3 | Prosthion | Most anterior point of the maxillary alveolar process in the midline |
| 4 | Basion | Most inferior point of the midpoint of the anterior curvature of the foramen magnum |
| 5 | Opisthion | Most inferior point of the midpoint of the posterior curvature of the foramen magnum |
| 6 | Zygomatic Arch R | Most latero-inferior point on the zygomaticotemporal suture R |
| 7 | Jugale R | Intersection of the zygoma, maxilla and sphenoid R |
| 8 | Foramen infra-orbitale R | Midpoint on the superior margin of the infraorbital foramen R |
| 9 | Nasal cavity R | Latero-inferior point in the curvature of the nasal cavity R |
| 10 | Nasal cavity L | Latero- inferior point in the curvature of the nasal cavity L |
| 11 | Foramen infra-orbitale L | Midpoint of the superior margin of the infraorbital foramen L |
| 12 | Jugale L | Intersection of the zygoma, maxilla and sphenoid L |
| 13 | Zygomatic Arch L | Most latero-inferior point on the zygomaticotemporal suture L |
| 14 | Orbitale L | Most antero-inferior point on the inferior orbital margin L |
| 15 | Frontozygomatic suture L | Mid-anterior point on the lateral curve of the orbit on the intersection of the zygoma and frontal bone L |
| 16 | Supra-orbitale L | Anterolateral point of the supra-orbital notch of the supra-orbital rim L |
| 17 | Orbitale R | Most anteroinferior point on the inferior orbital margin R |
| 18 | Frontozygomatic suture R | Mid-anterior point on the lateral curve of the orbit on the intersection of the zygoma and frontal bone R |
| 19 | Supraorbitale R | Antero-lateral point of the supraorbital notch of the superior orbital rim R |
| 20 | Rhinion | Most antero-inferior point of the nasal bone |
| 21 | Pterion R | Most antero-inferior point of the parietal bone on the intersection of the frontal, parietal, and sphenoid bones R |
| 22 | External auditory meatus superior R | Most latero-superior point of the external auditory canal R |
| 23 | External auditory meatus inferior R | Most latero-inferior point of the external auditory canal R |
| 24 | Mastoid process R | Most inferior point of the mastoid process R |
| 25 | Pterion L | Most antero-inferior point of the parietal bone on the intersection of the frontal, parietal, and sphenoid bones R |
| 26 | External auditory meatus superior L | Most latero-superior point of the external auditory canal L |
| 27 | External auditory meatus inferior L | Most latero-inferior point of the external auditory canal L |
| 28 | Mastoid process L | Most inferior point of the mastoid process L |

**Supplementary Table 1.** Skull landmarks used for dense correspondence, with numbers referring to Supplementary Figure 1. L= left side; R = right side.


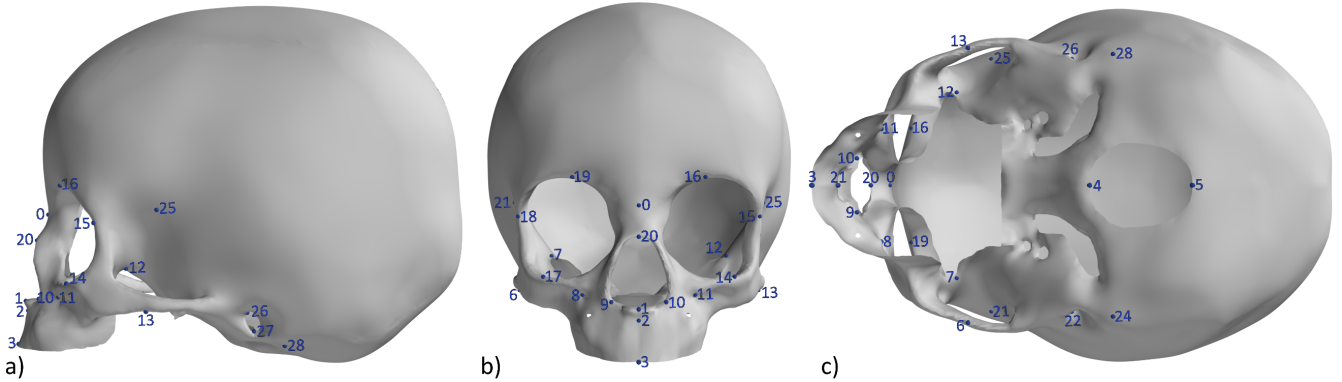


**Supplementary Figure 1:** Landmarks used to annotate the selected population skull reconstructions. Landmarks are shown on the template mesh.

## **“Stiffness Factor” for Dense Mesh Registration**


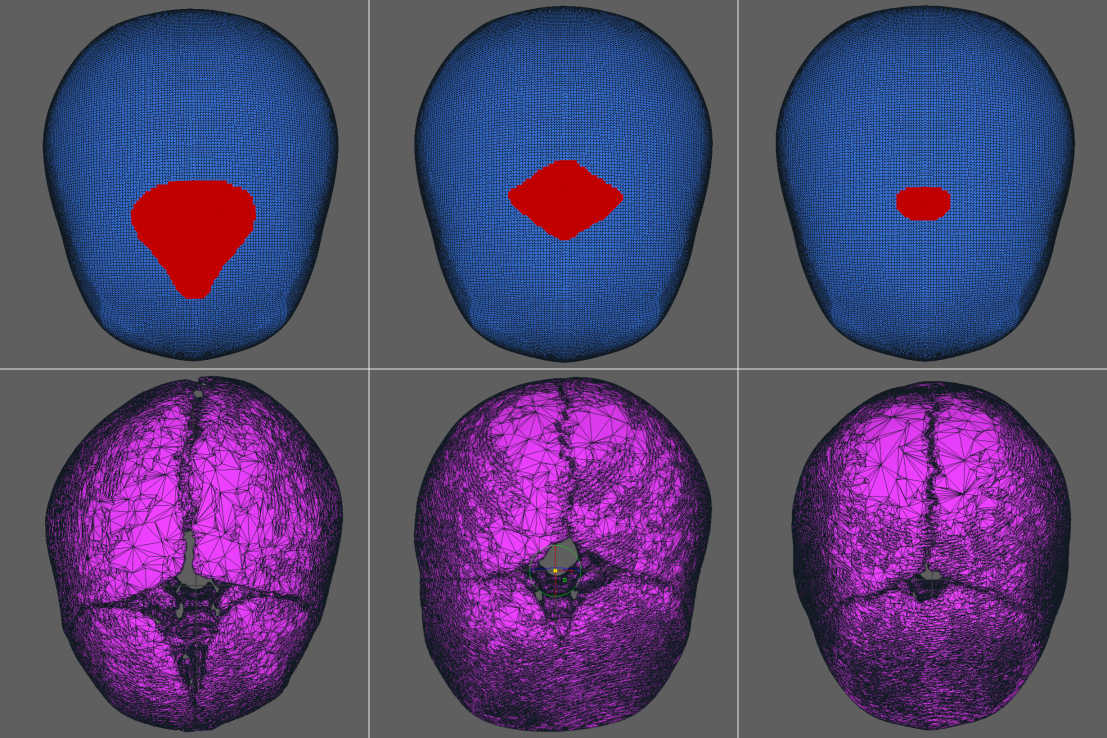


**Supplementary Figure 2:** “Stiffness factor” applied to prevent inward collapse of the template mesh (top row) in the region of an open anterior fontanelle during mesh registration. The size of the stiffness region (red) was adjusted depending on the size of the opening as shown.

## **Measurement Combination**

The combined means and standard deviations reported in this manuscript were collated from the paper of Waitzman et al [5]. To calculate the combined mean and standard deviation of two datasets with sample sizes of *n_1_* and *n_2_*, means of *µ_1_* and *µ_2_*, and standard deviations of *σ_1_* and *σ_2_*, respectively, the following formulae were applied:

$$\mu= \frac{n_{1}\mu_{1}+ n_{2}\mu_{2}}{n_{1}+n_{2}}$$

$$\sigma=\sqrt{\frac{n_{1}\sigma_{1}^{2}+ n_{2}\sigma_{2}^{2}+n_{1}\left( \mu_{1}-\mu\right)^{2}+n_{2}\left( \mu_{2}-\mu\right)^{2}}{n_{1}+n_{2}-1}}$$

Generalising this to an arbitrary number of datasets, *k*, yields:

$$\mu= \frac{\sum_{i=1}^{k} n_{i}\mu_{i}}{\sum_{i=1}^{k} n_{i}}$$

$$\sigma=\sqrt{\frac{\sum_{i=1}^{k} n_{i}\sigma_{i}^{2}+ \sum_{i=1}^{k} n_{i}\left( \mu_{i}-\mu\right)^{2}}{\sum_{i=1}^{k} n_{i}-1}}$$

## **Population Distribution for Gender and Age Groups**


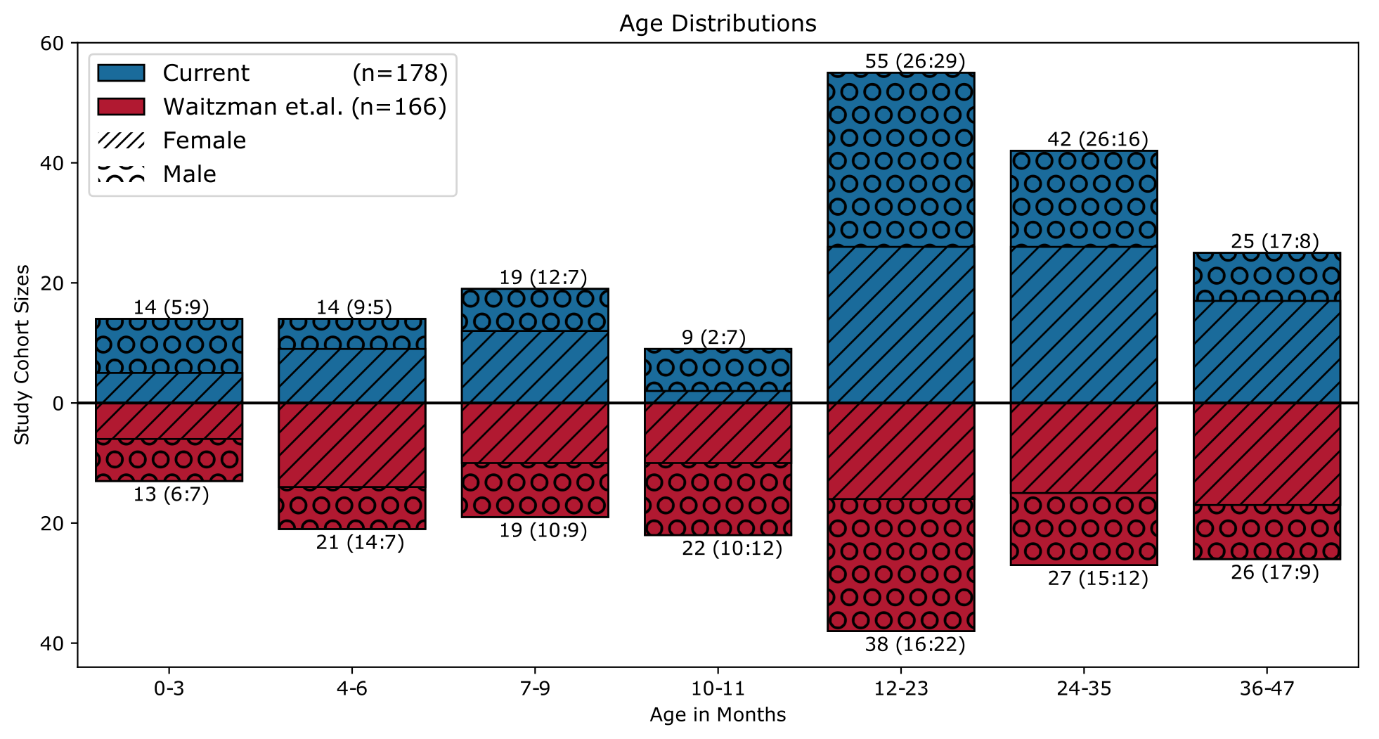


**Supplementary Figure 3:** Age distribution for the selected paediatric dataset (blue) and for the Waitzman et al. cohort (red) [5]. Annotations indicate the total number of samples in each age group, including the male:female gender ratio.
